# Breast cancer subtype of French women is not influenced by socioeconomic status: A population-based-study

**DOI:** 10.1371/journal.pone.0170069

**Published:** 2017-02-15

**Authors:** Aviane Auguste, Marion Cortet, Tienhan Sandrine Dabakuyo-Yonli, Ludivine Launay, Laurent Arnould, Isabelle Desmoulins, Patrick Roignot, Ariane Darut-Jouve, Marie-Laure Poillot, Aurélie Bertaut, Patrick Arveux

**Affiliations:** 1 Breast and Gynaecologic Cancer Registry of Côte d’Or, Georges-François Leclerc Comprehensive Cancer Care Centre, 1 rue du Professeur Marion, Dijon, France; 2 EA 4184, Medical School, University of Burgundy, 7 boulevard Jeanne d’Arc, Dijon, France; 3 Plateforme ERISC, U 1086 INSERM "Cancers and Preventions", François Baclesse Comprehensive Cancer Care Centre, 3 avenue du Général Harris, CAEN, France; 4 Pathology Centre, 33 rue Nicolas Bornier, Dijon, France; 5 Oncology Centre of Park, 18 cours du Général De Gaulle, Dijon, France; 6 Biostatistics Unit, Georges-François Leclerc Comprehensive Cancer Care Centre, 1 rue du Professeur Marion, Dijon, France; University of South Alabama Mitchell Cancer Institute, UNITED STATES

## Abstract

**Context:**

The molecular subtype of breast tumours plays a major role in cancer prognosis and treatment options. Triple negative tumours (TN) carry the worst prognosis and affects most frequently women of low socioeconomic status (SES). Studies have shown that non-biologic factors, such as the socioeconomic status could have an influence on tumour biology. To this date no study has been done investigating this association in French women. The objective is to study the association between the SES and the molecular tumour subtype of breast cancer patients in the French county of Côte d’Or. This study benefits from the population data from the Côte d’Or breast cancer registry known for its strict quality control policy.

**Methods:**

Invasive breast cancer cases between 2003 and 2013 were extracted from the Breast cancer registry database in Côte d’Or. A multivariate analysis was conducted using a hierarchical polytomous regression for the multinomial outcomes for the cancer subtype with HR+/HER2 as reference category.

**Results:**

A total of 4553 cases were included in our study. There was no significant association found between SES and tumour subtype in French women at diagnosis. Women older than 75 years were less likely to have a TN and HR+/HER2+ breast cancer (OR = 0.66; CI95% = [0.46–0.94] and OR = 0.51; CI95% = [0.37–0.70] respectively). Women with TN tumour subtype had significantly less lymph node invasion when compared to HR+/HER2- subtype (OR = 0.71; CI95% = [0.54–0.92]).

**Conclusion:**

No significant association was found between socioeconomic status and molecular subtype. Further studies are needed to clarify the mechanisms associated with developing each tumour subtype.

## Introduction

Breast cancer is the most common cancer among women in the world [1]. In France, about 50 000 women are diagnosed with breast cancer each year [2]. Breast tumours are classified into one of four clinically pertinent molecular subtypes based on the joint status of the hormone receptors (oestrogen and progesterone) and human epidermal growth factors receptor 2 (HER2) [3,4]. The hormone receptors (HR) and HER2 receptors are said to be positive if they are overexpressed in a tumour cell. The HR-positive tumours are the most common at diagnosis (80%), and the HER2-positive represents about 15–20% [5]. Receptor testing is readily available and performed routinely during breast cancer diagnosis in France. The information on receptors is of clinical importance since treatment options are selected based on the joint receptor status. Each subtype has its own distinct histological profile and risk factors [2]. The HER2+ tumours are known to be very aggressive and have poor survival in women [3]. Nowadays, the HR+/HER2+ subtype has a better prognosis, particularly in metastatic tumours because it has molecular targets for hormone therapy as well as other targeted treatments like Trastuzumab (Herceptin^TM^) [4]. Tumours which are negative for both hormone receptors (HR) and HER2 receptor are called triple negative tumours (TN). These TN tumours have the worst prognosis and affect more often underprivileged women in the USA [5,6]. Socioeconomic status influences the exposure to several risk factors which could modify tumour biology [7,8]. It has been shown that women in high poverty areas tend to secrete more oestrogen when compared to women with a more affluent socioeconomic status [8]. Few studies have looked at the association between socioeconomic status and tumour subtypes [9–14]. Among these studies, there have been registry based analyses where a significant association between the SES and the breast cancer subtype in American women was observed, in particular among the TN cases who had higher odds of being in the lowest socioeconomic stratum when compared to the other subtypes To this date no study has been done investigating this association in women residing in France. Our objective is to study the association between socioeconomic status and breast cancer subtype at diagnosis among women in the French county of Côte d’Or.

## Methods

### Study population

A population-based study was undertaken using data from the Côte d’Or breast and gynaecological cancer registry. This Breast and Gynaecological Cancer Registry is the only one in France that focuses on breast and gynaecological cancers. It has been collecting comprehensive population-based data since 1982 in this area located in the northeast of France. Women with primary invasive breast cancer and living in the rural county of Côte d’Or at the time of diagnosis were retrospectively selected from January 2003 to December 2013 to be included in this study. The year 2003 is the year that the registry began collecting systematically data for HER2. The data extraction which was performed was anonymized prior to reception of the data by the investigators and did not carry the patient’s names or personal information which could identify them (name initials). The registry has the necessary regulatory agreements to use the patient data from the National Commission on Informatics and Liberty (CNIL), aimed at ensuring the application of data privacy laws. (CNIL authorisation number DR-2012-038).

### Variables

The Socioeconomic status for each case was determined by using the French European Deprivation Index (FEDI) which is an ecological deprivation index, based on a European 2006 survey and French 2007 census data. This index was developed to take into account the socioeconomic and cultural particularities of the French context [15]. This index measures a deprivation score on an area-level for all the IRISs in France (“Ilots Regroupés pour l’Information Statistique”: Merged Islet for Statistical Information) which are geographic zones containing approximately 2000 inhabitants. The individuals were categorised into quintiles according to the deprivation score of the IRIS in which they live.

The cancer stage was determined using the American Joint Committee on Cancer Tumour Node Metastasis (AJCC TNM) classification of malignant tumours and cases were categorised into 4 classes (Stage I, II, III and IV) [16]. Hormone receptor and HER2 receptor status was determined using an immunohistochemistry exam. In case of an uncertain result for the HER2 receptor status, a FISH exam (Fluorescent *in situ* Hybridisation) was used to clarify the status. the molecular subtype was determined by combining the hormone receptor status and the HER2 receptor status. Individuals were classified into 4 categories: HR+/HER2-, TN (triple negative), HR-/HER2+ (HER2-Overexpressing) and HR+/HER2+. The lymph node status was classified as being either N0 for absence cancer cells found in any nearby nodes or N+ for presence of cancer cells in the lymph nodes regardless of the location. Women were categorised into 3 groups for the tumour size T0+T1 (size ≤ 2cm), T2 (size between 2 and 5cm) and T3+T4 (size>5cm or extension either directly to the chest wall or to the skin).

### Statistical analysis

Statistical analysis was performed using SAS 9.3 software (SAS Institute, Cary, NC, USA). A descriptive analysis was used to examine the demographic and clinical tumour characteristics and their distribution according to the 4 molecular breast cancer subtypes. A Chi-squared test was used to assess the association between the breast cancer subtypes and the different patient characteristics. Tests giving a p-value lower than 5% were considered to be significant. A multivariate analysis was conducted using a hierarchical polytomous regression for the multinomial outcomes for the cancer subtype and to take into account the aggregated data for the SES. This method was used to calculate the odds of having each of the subtypes compared with HR+/HER2-, after adjusting for the differences in age at diagnosis, tumour histoprognostic Scarff Bloom and Richardson (SBR) grade, tumour size and histological type in the model. Missing values were not included in our analysis and were omitted in the regression model. The class HR+/HER2- was selected as a reference class because this subtype is regarded as having a good prognosis in relation to the other subtypes [17].

## Results

### Patient characteristics

**Fig 1:**
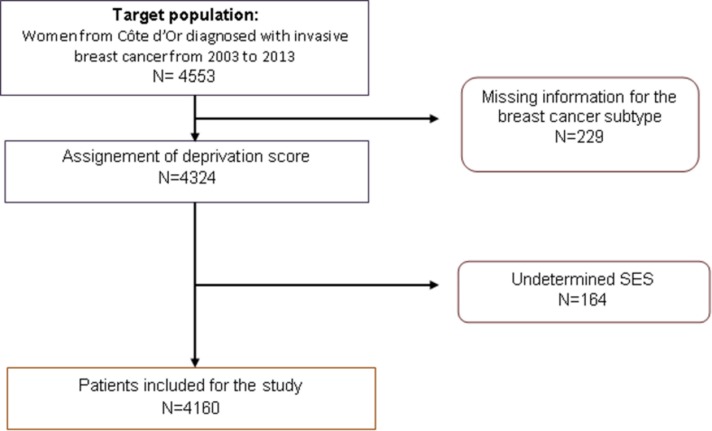
Flow chart for the selection of subject for the statistical analysis.

**Fig 2:**
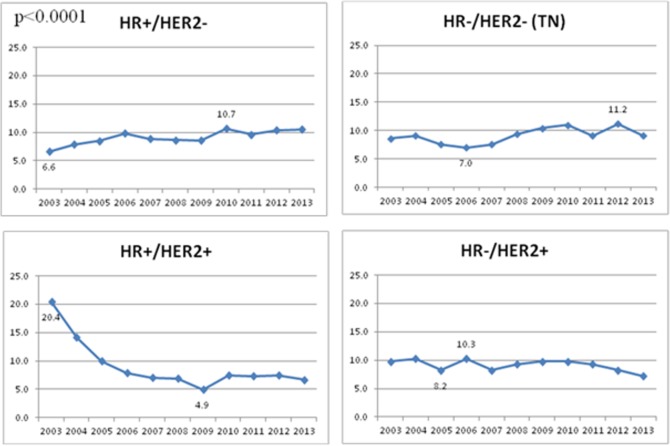
Distribution of breast cancer diagnosis by tumour subtype and year of diagnosis.

From 2003 to 2013, 4553 invasive breast cancer cases in the Côte d’Or breast cancer registry were eligible for the study. After extraction of our data, 229 cases had an undetermined molecular subtype due to missing data one or more of the receptors (HR or HER2). The remaining cases were then assigned a deprivation score using their address and the FEDI. The deprivation score for 164 cases was not able to be determined and were then omitted from the analysis due to lack of deprivation information on their place of residence. Our final analysis was carried out on 4160 women (Fig 1). Among the cases, HR+/HER2- were the predominant subtype and represented 70.1% of the total study population (3192 cases). This subtype is followed by the subtype HR+/HER2+ representing 11.6% (528 cases). The TN cases accounted for 8.9% (403 cases), and 4.4% of the cases were the HER2-Overexpressing subtype (201 cases). Table 1 provides a description of the sociodemographic and clinical characteristics of the patients by breast cancer subtype. The younger patients (<50 years) were found to have more frequently the TN subtype (11.7%), HR+/HER2+ (15.1%) and HER2-oOverexpressing (5.7%) subtypes when compared with the older patients. TN cases were significantly more likely to have high grade tumours (p< 0.0001). Similarly the earlier tumour stages had significantly higher proportions for the HR+/HER2- subtype compared to the other stages. In addition, the SBR grade I tumours were mainly found to be HR+/HER2- cases (85.7%) and there was a substantial amount of grade III tumours which were TN (28.1%). The tumours of size T3 (>5cm) were found to be more frequently TN cases (14.3%) compared to smaller tumours. A significantly greater proportion of the HR+/HER2+ cases were diagnosed during the first half of our study period (2003–2007) (p< 0.0001) (Fig 2).The least affluent SES quintile (Q1) represents 30% of women. Women with HER2-Overexpressing tumours were found to belong more frequently to the most affluent SES quintile (Q5) when compared to the other subtypes. Women with TN tumours were found the most frequently in middle SES quintile.

**Table 1:** Characteristics of breast cancer subtypes of patients in Côte d’Or selected for the study (2003–2013).

Variable	Category	Total		HR+/HER2-		HR-/HER2- (TN)		HR+/HER2+		Her2-overexpressing		Unknown		P-value
		n = 4553		n = 3192 (70.1)		n = 403 (8.9)		n = 528 (11.6)		n = 201 (4.4)		n = 229 (5.0)		
		n (col %)^a^		n (row %)^b^		n (row %) ^b^		n (row %) ^b^		n (row %) ^b^		n (row %) ^b^		
Age group														< .0001
	< 50	1057	(23.2)	678	(64.1)	124	(11.7)	160	(15.1)	60	(5.7)	35	(3.3)	
	50–74	2521	(55.4)	1837	(72.9)	192	(7.6)	290	(11.5)	106	(4.2)	96	(3.8)	
	75+	975	(21.4)	677	(69.4)	87	(8.9)	78	(8.0)	35	(3.6)	98	(10.1)	
Diagnosis year														< .0001
	≤ 2007	1983	(43.6)	1318	(66.5)	161	(8.1)	314	(15.8)	95	(4.8)	95	(4.8)	
	≥ 2008	2570	(56.5)	1874	(72.9)	242	(9.4)	214	(8.3)	106	(4.1)	134	(5.2)	
Histopronostic SBR grade														< .0001
	I	1312	(28.8)	1124	(85.7)	19	(1.5)	90	(6.9)	5	(0.4)	74	(5.6)	
	II	2140	(47.0)	1618	(75.6)	102	(4.8)	270	(12.6)	65	(3.0)	85	(4.0)	
	III	950	(20.9)	401	(42.2)	267	(28.1)	155	(16.3)	113	(11.9)	14	(1.5)	
	Missing	151	(3.3)	49		15		13		18		56		
Metastasis														0.4218
	Yes	258	(5.7)	172	(66.7)	30	(11.6)	32	(12.4)	16	(6.2)	8	(3.1)	
	No	4231	(92.9)	2993	(70.7)	369	(8.7)	496	(11.7)	183	(4.3)	190	(4.5)	
	Missing	64	(1.4)	27		4		0		2		31		
Node status														< .0001
	N0	2786	(61.2)	2002	(71.86)	225	(8.08)	289	(10.4)	96	(3.5)	174	(6.3)	
	N+	1721	(37.8)	1178	(68.5)	175	(10.2)	233	(13.5)	103	(6.0)	32	(1.9)	
	Missing	46	(1.0)	12		3		6		2		23		
SES														0.2758
	1 (low)	1370	(30.1)	979	(71.5)	118	(8.6)	162	(11.8)	50	(3.7)	61	(4.5)	
	2	632	(13.9)	439	(69.5)	56	(8.9)	76	(12.0)	34	(5.4)	27	(4.3)	
	3	808	(17.8)	559	(69.2)	82	(10.2)	90	(11.1)	28	(3.5)	49	(6.1)	
	4	820	(18.0)	558	(68.1)	68	(8.3)	107	(13.1)	41	(5.0)	46	(5.6)	
	5 (high)	747	(16.4)	536	(71.8)	60	(8.0)	75	(10.0)	42	(5.6)	34	(4.6)	
	Missing	176	(3.9)	121		19		18		6		12		
Stage (TNM)														< .0001
	I	2134	(46.9)	1586	(74.3)	137	(6.4)	227	(10.6)	63	(3.0)	121	(5.7)	
	II	1185	(26.0)	804	(67.9)	126	(10.6)	156	(13.2)	57	(4.8)	42	(3.5)	
	III	903	(19.8)	602	(66.7)	104	(11.5)	111	(12.3)	63	(7.0)	23	(2.6)	
	IV	258	(5.7)	172	(66.7)	30	(11.6)	32	(12.4)	16	(6.2)	8	(3.1)	
	Missing	73	(1.6)	28		6		2		2		35		
Tumour size														< .0001
	T1+ T0	2874	(63.1)	2106	(73.3)	190	(6.6)	341	(11.9)	95	(3.3)	142	(4.9)	
	T2	1214	(26.7)	800	(65.9)	149	(12.3)	140	(11.5)	77	(6.3)	48	(4.0)	
	T3+ T4	440	(9.7)	280	(63.6)	63	(14.3)	46	(10.5)	26	(5.9)	25	(5.7)	
	Missing	25	(0.6)	6		1		1		3		14		
Tumour type														< .0001
	Ductal	3648	(80.1)	2500	(68.5)	367	(10.1)	459	(12.6)	189	(5.2)	133	(3.7)	
	Lobular	603	(13.2)	507	(84.1)	12	(2.0)	52	(8.6)	3	(0.5)	29	(4.8)	
	Other	217	(4.8)	161	(74.2)	20	(9.2)	9	(4.2)	4	(1.8)	23	(10.6)	
	Missing	85	(1.9)	24		4		8		5		44		

a: percentage of women from the total study population (4553).

b: percentage of women from total for a given characteristic.

### Multivariate analysis

Table 2 shows the odds ratios for hierarchical multivariate polytomous regression analysis with HR+/HER2- as the reference category. No significant association was observed between SES and tumour subtype (p = 0.3956), However, we found that women older than 75 years are less likely to have a TN and HR+/HER2+ breast cancer (OR = 0.67; CI95% = [0.47–0.96]) and OR = 0.52; CI95% = [0.37–0.72] respectively). Women with lobular tumours were more significantly likely to be diagnosed with HR+/HER2- when compared to the other subtypes, in particular the TN (OR = 0.24; CI95% = [0.13–0.46]) and HER2-Overexpressing tumours (OR = 0.12; CI95% = [0.04–0.38]). Regarding the histopronostic SBR grade, HR negative subtypes (TN and HER-Overexpressing) versus HR+/HER2- had considerably greater odds to be grade III (TN OR = 40.06; CI95% = [23.71–67.71], and HER2-Overexpressing OR = 47.80; CI95% = [19.00–120.23]). This association was found to be less intense for the HR+/HER2+ subtype (OR = 4.59; CI95% = [3.37–6.26]). The odds of having TN and HER2-Overexpressing subtype versus HR+/HER2- doubles when the tumour is bigger than 5cm (T3+T4) compared to tumours 2cm or under (T0+T1) (OR = 2.27; CI95% = [1.19–4.33]). Regarding lymph nodes status, women with TN tumour subtype have significantly more N0 tumours when compared to HR+/HER2- subtype (OR = 0.71; CI95% = [0.54–0.92]). The odds of being diagnosed with the HR+/HER2+ subtype was significantly greater between 2003 and 2007 compared to the second half of our study period (OR = 1.99; CI95% = [1.63–2.43]).

**Table 2:** Estimated odds ratios (OR) with 95% confidence intervals (CI) from hierarchical polytomous regression models of association between patient sociodemographic and clinical characteristics with breast cancer subtype (2003–2013).

Variable	Category	HR+/HER2-		HR-/HER2- (TN)			HR+/HER2+			HR-/HER2+ (HER2-Overexpressing)			p value
		n = 2992 (74.4%)		n = 367 (9.1%)			n = 487 (12.1%)			n = 174 (4.3%)			
		n	Reference	n	OR	(95% CI)	n	OR	(95% CI)	n	OR	(95% CI)	
Age group													0.0023
	< 50	611		110	1		137	1		48	1		
	50–74	1770		182	0.75	(0.57–1.00)	283	0.80	(0.64–1.01)	94	0.92	(0.63–1.35)	
	75+	611		75	0.67	(0.47–0.96)	67	0.52	(0.37–0.72)	32	0.63	(0.39–1.05)	
Diagnosis year													< .0001
	≤ 2007	1247		147	0.80	(0.63–1.02)	291	1.99	(1.63–2.43)	82	1.09	(0.79–1.51)	
	≥ 2008	1745		220	1		196	1		92	1		
Histopronostic SBR grade													< .0001
	I	1071		17	1		86	1		5	1		
	II	1547		99	4.32	(2.54–7.33)	253	2.11	(1.60–2.71)	59	7.74	(3.07–19.60)	
	III	374		251	40.06	(23.71–67.71)	148	4.59	(3.38–6.32)	110	47.80	(19.00–120.23)	
Node status													0.0043
	N0	1898		211	1		270	1		84	1		
	N+	1094		156	0.71	(0.54–0.92)	217	1.25	(1.01–1.55)	90	1.05	(0.74–1.48)	
SES													0.3956
	1 (low)	952		112	1.13	(0.78–1.63)	154	1.21	(0.89–1.66)	45	0.75	(0.47–1.19)	
	2	429		51	1.21	(0.78–1.87)	73	1.32	(0.92–1.90)	30	1.16	(0.69–1.97)	
	3	545		78	1.29	(0.87–1.92)	85	1.17	(0.82–1.65)	24	0.64	(0.37–1.11)	
	4	545		66	1.08	(0.72–1.62)	102	1.38	(0.99–1.94)	37	0.98	(0.60–1.61)	
	5 (high)	521		60	1		73	1		38	1		
Tumour size													0.00295
	T1+ T0	2002		173	1		317	1		75	1		
	T2	744		143	1.29	(0.97–1.70)	131	0.85	(0.65–1.08)	73	1.48	(1.02–2.15)	
	T3+ T4	246		51	2.27	(1.19–4.33)	39	0.81	(0.42–1.55)	26	2.22	(1.00–5.15)	
Tumour type													< .0001
	Ductal	2360		342	1		428	1		168	1		
	Lobular	485		11	0.24	(0.13–0.46)	50	0.67	(0.49–0.93)	3	0.12	(0.04–0.38)	
	Other	147		14	1.45	(0.76–2.74)	9	0.51	(0.26–1.02)	3	0.68	(0.21–2.24)	

## Discussion

In light of previous studies [9–14], we suspected a possible association between the molecular breast cancer subtype and the SES among French women in the rural county of Côte d’Or. Our findings suggest an absence of such an association. However, in our study age was found to be a factor associated with the tumour subtype. Women below the age of 50 were more at risk for TN and HR+/HER2+ tumours. This finding coincides with results found in a previous study [10]. Women with SBR grade III tumours were found to be at a considerably much higher risk of being diagnosed with HR-negative tumours when compared to lower grade tumours. The positive association between tumour grade and tumour subtype is in agreement with the literature:. Moreover, this elevated risk of HR-negative tumours could be explained by the environmental exposures and lifestyle differences between women living in France and those observed in other studies. Lymph node involvement for the TN tumours follows the trends in other studies [18,19]. The frequency of the tumour type (lobular and ductal) is consistent with the literature and lobular tumours were associated with a substantial reduction in the risk of being diagnosed with HR-negative tumours (TN and HER2-Overexpressing) [20–22]. A strong association was found between the molecular subtype and the year of diagnosis. This trend was found to be most prominent in the HR+/Her2+ subtype with a significantly larger proportion of cases occurring in the first half of the study period. This is due to a temporary random misclassification issue encountered during 2003 and 2004 caused by an inadequately adopted procedure for receptor testing in certain pathology laboratories.

In our study, the overall SES of the cases was not found to be associated with the molecular subtype at diagnosis. This finding opposes our initial hypothesis that socioeconomic related factors such as obesity and parity led to an alteration of tumour biology through an increase in hormone secretion. The lack of an association between SES and tumour subtype is consistent with a study which shows that the incidence rate of HR-negative breast cancer subtype (TN and HER2-Overexpressing) is independent of SES [9]. In contrast, A study has reported that low SES is significantly associated with an increased risk for the TN subtype [6]. These findings suggest that non-genetic and non-biological factors in the low SES environment alter the tumour biology of these patients (parity, low physical activity) [23–26]. This variation in results could be explained by the different confounding factors taken into account in the various reports.

Some reports found an association between ethnicity and tumour subtype. This association persisted even after controlling for differences in SES [10–13,27]. A similar report studying ethnicity, SES and tumour subtype showed contrasting results. It was shown that low SES was associated with a higher prevalence of HR-negative cancers despite the race/ethnicity of the individual [14]. The Côte d’Or breast cancer registry is not authorised to collect data on patient race and ethnicity therefore this information was not available for the study. However, Côte d’Or is a French department known to have a large Caucasian population and thus the bias is kept to a minimum.

Obesity is a major factor associated with high poverty areas. Studies where an association between SES and subtype was observed, showed that obesity and dietary habits play big role in hormone receptor status [28–30]. In obese women, there is an increase in insulin secretion in the bloodstream which provokes inflammatory reactions resulting in an increase in cytokines and tumour necrosis factor (TNF) and the Tumour growth factor β (TGF-β). The latter disturbs the genomic stability through the activation of cell-signalling pathways involved in cell proliferation such as the PI3K-AKT pathway [31–33]. As a result, obesity has been identified as the key component behind the association between SES and tumour subtype. We would have liked to take into account this factor in our study but we lacked information on individual risk factors (reproductive history and breast feeding, parity, diet, body mass index, alcohol use, oral contraceptives and hormone replacement treatments) for breast cancer and therefore we were not able to adjust on them in our regression model. A study found an association between SES and molecular subtypes. This association could be explained by differences in lifestyles of the women of different socioeconomic strata [14], whereas other reports suggest that this association is due to innate biological and genetic differences among women with a very pronounced difference between black and white women [34,35].

There are several contradictory findings in the literature concerning the association between SES and breast cancer subtype. Further research is required to confirm these hypotheses. In particular, ethnicity and other risk factors like body fat need to be taken into consideration when evaluating the impact of SES.

We acknowledge that our study has some limitations that need to be considered when interpreting the data. First, it is subject to all the inherent biases associated with its retrospective design. However, these biases are limited as we had very few missing data as a breast cancer registry. Secondly, The French EDI is an ecological index used as a proxy for individual SES, and could potentially assign incorrect SES for a given individual. However, studies have shown that in France there is a correlation between the socioeconomic characteristics measured on the individual level and the IRIS level [36]. In addition, IRISs are very small geographic units (approximately 2000 inhabitants) which therefore lessen the ecological bias in our study. The smaller the geographic zone, the greater the accuracy of SES measures will be.

Finally, the major, the strength of our study lies in the use of population-based data of the Côte d’Or breast cancer registry and its extensive availability for specific tumour data like the hormone receptors and HER2 receptor status. The registry respects a strict data quality control policy which includes regular checking to ensure the complete follow-up for each patient and the use of crossing information sources. The comprehensiveness and the great quality of our data ensure that there is no selection bias, contrary to clinical trials or hospital cohorts in which patients are highly selected. Our population could therefore be considered representative of French women.

## Conclusion

In conclusion, despite the adjustment of socio-demographic (age) and tumour clinical characteristics, there was no significant association found between SES and tumour subtype in French women at diagnosis of breast cancer. In light of the contradictory finding in previous studies, further studies are needed to clarify the mechanisms in the populations where this association was found significant. Special consideration should be given to confounding factors and the use of individual socioeconomic data.
